# Acute autoimmune transverse myelitis following COVID-19 vaccination: A case report

**DOI:** 10.1097/MD.0000000000028423

**Published:** 2021-12-23

**Authors:** Satoshi Hirose, Makoto Hara, Kento Koda, Naotoshi Natori, Yuki Yokota, Satoko Ninomiya, Hideto Nakajima

**Affiliations:** Division of Neurology, Department of Internal Medicine, Nihon University School of Medicine, Tokyo, Japan.

**Keywords:** autoimmune, COVID-19, oligoclonal bands, transverse myelitis, vaccine

## Abstract

**Rationale:**

Transverse myelitis is an infectious or noninfectious inflammatory spinal cord syndrome. We report a rare case of transverse myelitis following vaccination against COVID-19.

**Patient concerns:**

A 70-year-old male presented with progressive sensorimotor dysfunction of the bilateral lower limbs 7 days after receiving the mRNA-1273 vaccine against COVID-19. Spinal magnetic resonance imaging revealed intramedullary lesions with gadolinium enhancement on the Th1/2 and Th5/6 vertebral levels. Cerebrospinal fluid (CSF) testing showed a mildly increased level of total protein and positive oligoclonal bands (OCB).

**Diagnosis:**

The patient was diagnosed with acute transverse myelitis.

**Intervention:**

The patient received 5 days of intravenous methylprednisolone pulse (1000 mg/day) followed by oral prednisolone (30 mg/day with gradual tapering).

**Outcomes:**

The patient fully recovered from muscle weakness of the lower limbs. He was discharged from our hospital and able to independently walk without unsteadiness.

**Lesson:**

This is a rare case of transverse myelitis following COVID-19 vaccination. Positive OCB in CSF in the present case highlights the possibility of autoimmune processes, including polyclonal activation of B lymphocytes, following vaccination.

## Abstract 2

Supplemental Digital Content is available in the text

## 1 Introduction

Transverse myelitis is an infectious or noninfectious inflammatory spinal cord syndrome, which develops sensory, motor, or autonomic dysfunction bilaterally.^[1]^ Although the prognosis for a patient to regain function after transverse myelitis is highly dependent on its etiology, 50% to 70% of patients achieve at least partial recovery and ability to walk.^[1]^ Reports of postvaccination transverse myelitis cases,^[2]^ described onsets of the disease ranging from 2 days to 3 months after vaccination against hepatitis B virus, measles–mumps–rubella, diphtheria–tetanus–pertussis, and others. Recently, several cases of transverse myelitis from 1 to 14 days after COVID-19 vaccination have been also reported.^[3–9]^ Here we report a rare case presenting with transverse myelitis following COVID-19 vaccination with mRNA-1273, Moderna.

## 2 Case presentation

A 70-year-old Japanese male was admitted to our hospital with bilateral lower extremities hypoesthesia and mild paraparesis, which had emerged 17 days before admission. The patient had received the first dose of COVID-19 vaccination with mRNA-1273, Moderna, 24 days before admission, without any acute side effect. The patient had a medical history of hypertension, hyperuricemia, and alcoholic liver cirrhosis, and amlodipine, spironolactone, benzbromarone, rifaximin, lactitol, vonoprazan, and ursodeoxycholic acid were administered. His history was otherwise unremarkable.

A neurological examination revealed that he was alert and well oriented without aphasia and had no neurological involvement of the cranial nerves. He exhibited severely impaired bilateral perceptions to pinprick (predominantly on the left side), vibration (predominantly on the right side) below the level of the eighth thoracic dermatome, and mild weakness of the bilateral lower limbs (MRC 4/5) (predominantly on the right side). He also showed hyperreflexia of the bilateral lower limbs and bilateral Babinski signs. We did not observe any bladder and rectal disturbance or fever and any other signs of systemic infection.

**Figure 1:**
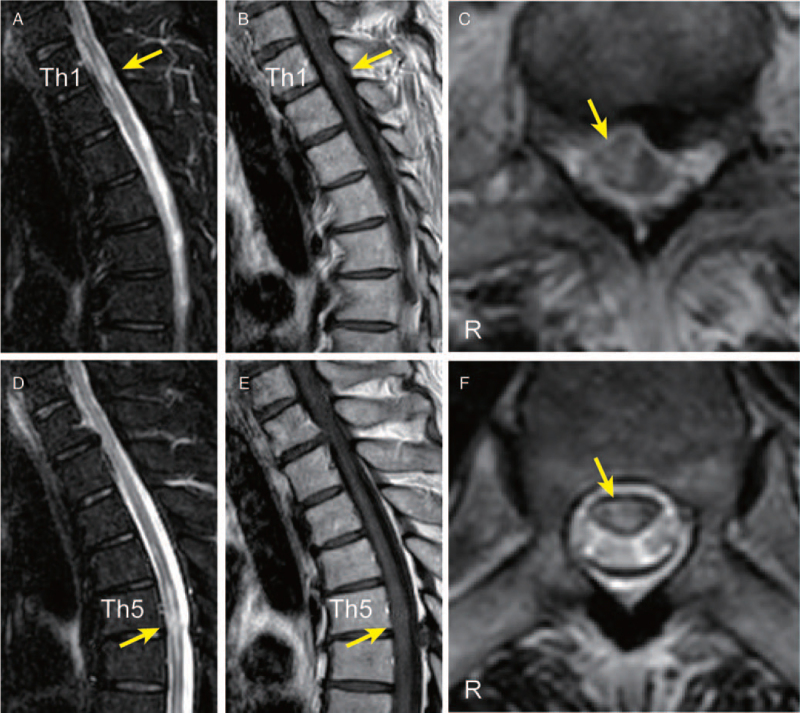
Spinal MRI. (A) Sagittal section of T2-weighed image of cervicothoracic spine shows a high-intense area located in the Th1/2 vertebral level. (B) Gadolinium-enhanced T1-weighted image shows focal weak gadolinium enhancement in the T2 high-intense area. (C) Transverse T2-weighted image shows a high-intense area in the Th1/2 vertebral level. (D–F) Other sections of the same sequence as panels A–C show another high-intense area located in the Th5/6 vertebral level.

**Figure 2:**
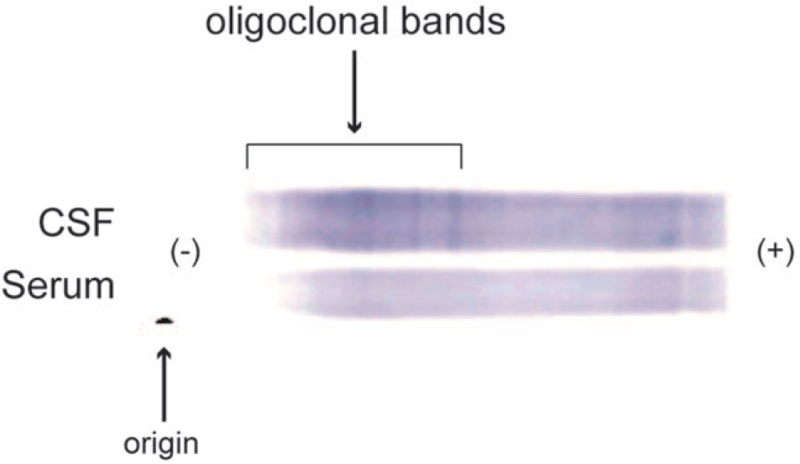
Oligoclonal bands patterns. More negatively-charged IgG molecules migrate further to the anode (right side), whereas less negatively-charged IgG molecules remain closer to the cathode (left side). The bands are present only in cerebrospinal fluid and not in serum.

Spinal magnetic resonance imaging (MRI) revealed multiple high-intense areas on a T2-weighted image located at the Th1/2 and Th5/6 vertebral levels with weak gadolinium enhancement (Fig. 1). On cranial MRI, we observed some non-specific lesions, which showed normal in the apparent diffusion coefficient mapping, on a T2-weighted image without any gadolinium enhancement. Electroencephalogram on admission was unremarkable. The cerebrospinal fluid (CSF) pressure was 90 mm H_2_O, and a cerebrospinal fluid (CSF) test showed a normal white blood cell count (1 cell/μL) and an increased level of total proteins (52 mg/dL). The albumin quotient (Q_Alb_: CSF albumin/serum albumin) was within the normal limit (8.0).^[10]^ We also detected positive results for the oligoclonal band (OCB) (Fig. 2), and both of myelin basic protein (MBP) and IgG index were within normal limits. Both the HSV- and VZV-DNA high-sensitive polymerase chain reactions with the CSF were negative. Regarding autoimmune encephalitis, we detected no autoantibodies against intracellular (Amphiphysin, Hu, Yo, CV2, Ri, Ma2/Ta, recoverin, Tr, GAD65, and others) and neuronal surface antigens (NMDAR, AMPAR, GABAbR, LGI1, Casper2, IgLON5, and DPPX) related to the central nervous system diseases measured by line blots (EUROLINE, Euroimmun, Lübeck, Germany) and cell-based assays (CBA) (BIOCHIP, Euroimmun, performed by Labor Berlin). Additionally, we performed in-house assays that included indirect immunolabeling with rat frozen brain sections and live primary hippocampal neurons,^[11,12]^ which revealed no anti-neuronal autoantibodies in the CSF. We detected no antibodies against aquaporin-4 and myelin oligodendrocyte glycoprotein using CBA in the serum and CSF. Our tests for autoantibodies of the systemic autoimmune diseases, including antinuclear antibodies, anti-dsDNA, antiphospolipid antibodies, SSA/SSB antibodies, ANCA, were all negative. Neither antibodies to HIV nor HTLV-1 was detected in serum, the rapid plasma reagin test was negative, and the severe acute respiratory syndrome coronavirus 2 (SARS-CoV-2) RNA test with nasopharyngeal swab was negative. Antibodies against SARS-CoV-2 were detected in serum; SARS-CoV-2 IgG was 55.6 AU/mL. After considering the differential diagnoses (Supplemental Digital Content Table S1, http://links.lww.com/MD/G552) in cases of OCB detected in CSF,^[13]^ we eventually diagnosed the patient with acute autoimmune transverse myelitis.

We treated the patient with 5 days of intravenous methyl-prednisolone pulse (IVMP) (1000 mg per day) followed by oral prednisolone (30 mg per day with gradual tapering). Fifty-seven days after onset of the symptoms, the patient fully recovered from the muscle weakness of the lower limbs without any adverse effect, while still experiencing mild hypoesthesia of vibration sensory signals. On day 34 of admission, when the patient independently walked without unsteadiness, he was discharged from our hospital. The patient provided written informed consent for publication of this case report.

## 3 Discussion

We have described the case of acute transverse myelitis, presenting with progressive sensorimotor dysfunction on the bilateral lower limbs, which developed 7 days after the mRNA-1273 (Moderna) COVID-19 vaccine; the neurological manifestation was substantially reduced with the treatment of corticosteroids. CSF testing revealed positive OCB in the present case. OCB is typically detected in various inflammatory conditions of the central nervous system (CNS), paraneoplastic disorders, and other conditions.^[13]^ After adequate exclusion of other causes (Supplemental Digital Content Table S1, http://links.lww.com/MD/G552) including CNS infection, systemic inflammation, paraneoplastic disorders, degenerative disorders, or functional disorders, we concluded the present case to be acute autoimmune transverse myelitis.

Postvaccination transverse myelitis cases have been reported from 2 days to 3 months after vaccination against hepatitis B virus, measles–mumps–rubella, diphtheria–tetanus–pertussis, and others.^[2]^ Recently, several cases of transverse myelitis from 1 to 14 days after COVID-19 vaccination have been also reported.^[3–9]^ A single case of transverse myelitis following the mRNA-1273 COVID-19 vaccine was recently reported by Khan et al^[8]^ Notably, the present case showed several differences from the previous case, including:

1. a longer delay between vaccination and onset (7 days in the present case vs 1 day in Khan et al case);
2. detection of OCB in the CSF;
3. and favorable outcomes with IVMP alone.

The mechanisms through which vaccines may induce transverse myelitis are unclear. However, Agmon-Levin et al speculated that vaccination may evoke an autoimmune response linked to transverse myelitis through 3 mechanisms^[2]^:

1. molecular mimicry between infectious antigens and self-antigens^[14]^;
2. acceleration of an ongoing autoimmune process by local activation of antigen–presenting cells and over processing of antigens by invading antigens^[15]^; and
3. polyclonal activation of B lymphocytes^[16]^ or bystander activation, which enhances cytokine production and further induces the expansion of autoreactive T-cells.^[17]^

Interestingly, the present case showed OCB only in the CSF, not in the serum (Fig. 2). This OCB pattern was termed “type 2” by Freedman et al and is considered to be indicative of intrathecal IgG synthesis.^[18]^ The absence of increased blood–brain barrier (BBB) permeability in the present case, as revealed by normal Q_Alb_,^[10]^ also supports the idea of intrathecal IgG synthesis. The immune system requires B lymphocytes to synthesize IgG; intrathecally synthesized IgG implies activation of certain clones of B lymphocytes.^[13]^ Therefore, positive OCB in the present case supports the idea that the third mechanism proposed by Agmon-Levin et al,^[2]^ namely polyclonal activation of B lymphocytes that enhances cytokine production and induces expansion of autoreactive T-cells, led to transverse myelitis. With respect to association of mRNA-based vaccine with autoimmunity, there is a concept of nucleic acid sensing as putative mechanism for autoimmune disease, as these mechanisms may also be involved in processing mRNA-based vaccine.^[19]^ It is also surprising that vaccination breakthroughs occur after 4.5 months, which is the time span the innate immune system is boosted after nucleic acid sensing started.^[20]^ In unveiling the autoimmune mechanisms underlying the neurological adverse events following the vaccine, there is an urgent need to accumulate, and synthesize into cohesive evidence, the cases of patients who developed transverse myelitis and other autoimmune-mediated disorders following the vaccine.

## 4 Conclusion

We presented a case of acute transverse myelitis following an mRNA-based COVID-19 vaccine. Positive OCB in the CSF in the present case highlights the possibility of autoimmune processes, including polyclonal activation of B lymphocytes, following vaccination. Although the association between the vaccine and transverse myelitis is unclear, it is important to bear in mind the possibility that the mRNA-based vaccine may induce transverse myelitis, probably related to autoimmune mechanisms.

## Author contributions

**Conceptualization:** Satoshi Hirose, Makoto Hara, Hideto Nakajima.

**Data curation:** Satoshi Hirose, Makoto Hara, Kento Koda, Naotoshi Natori, Yuki Yokota, Satoko Ninomiya, Hideto Nakajima.

**Funding acquisition:** Makoto Hara.

**Supervision:** Makoto Hara, Satoko Ninomiya, Hideto Nakajima.

**Writing – original draft:** Satoshi Hirose.

**Writing – review & editing:** Makoto Hara, Kento Koda, Naotoshi Natori, Yuki Yokota, Satoko Ninomiya, Hideto Nakajima.
